# Wholly Endoscopic Permeatal Removal of a Petrous Apex Cholesteatoma

**DOI:** 10.1155/2014/184230

**Published:** 2014-12-07

**Authors:** Todd Kanzara, Jagdeep Singh Virk, Sanjiv Chawda, Anthony O. Owa

**Affiliations:** ^1^ENT Department, Queen's Hospital, Barking, Havering and Redbridge University Hospitals NHS Trust, UK; ^2^Radiology Department, Queen's Hospital, Barking, Havering and Redbridge University Hospitals NHS Trust, UK

## Abstract

We report a case of a petrous apex cholesteatoma which was managed with a wholly endoscopic permeatal approach. A 63-year-old Caucasian male presented with a 10-year history of right-sided facial palsy and profound deafness. On examination in our clinic, the patient had a grade VI House-Brackmann paresis, otoscopic evidence of attic cholesteatoma behind an intact drum, and extensive scarring of the face from previous facial reanimation surgery. Imaging review was suggestive of petrous apex cholesteatoma. An initial decision to manage the patient conservatively was later reviewed on account of the patient suffering recurrent epileptic seizures. A wholly endoscopic permeatal approach was used with successful outcomes. In addition to the case report we also provide a brief description of the technique and a review of the relevant literature.

## 1. Introduction

Petrous bone cholesteatomas are slow growing epidermoid cysts arising from squamous cells in the petrous part of the temporal bone [1, 2]. They can be classified as supralabyrinthine, infralabyrinthine, massive labyrinthine, infralabyrinthine-apical, and apical [1]. Petrous bone cholesteatomas account for between 4% and 9% of all petrous bone lesions [3]. They can be congenital or acquired [1, 2]. A propensity to extend into the petrous apex, skull base, and internal auditory canal is well recognised. Furthermore, these lesions may involve other vital soft tissue structures such as the sigmoid sinus, jugular vein and artery, and the cerebellopontine angle [1, 2].

Petrous apex cholesteatomas (PACs) can present with hearing loss and a facial palsy with the geniculate ganglion being the most frequently affected part of the facial nerve. Other common presenting features include vertigo, tinnitus, otorrhoea, and otalgia [1, 4].

The inherent complexity of the anatomy of the petrous part of the temporal bone allied with the ability of cholesteatomas to involve other structures makes surgical extirpation less than straightforward. As such, numerous techniques which include the permeatal, transcochlear, transotic, transsphenoidal, and the lateral transtemporal approaches have been developed to reduce both intra- and postoperative complications [1, 2, 4, 5].

We present a case of a petrous apex cholesteatoma that was managed using a wholly endoscopic permeatal approach by the senior author.

## 2. Case Report

**Figure 1:**
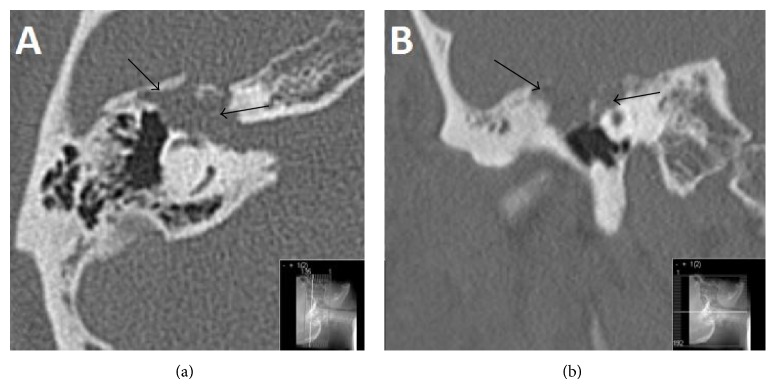
Axial (a) and coronal (b) CT images demonstrating a destructive abnormality (*black arrows demarcating extent*) involving the right geniculate ganglion extending into the right attic and eroding the bone around the right cochlea.

**Figure 2:**
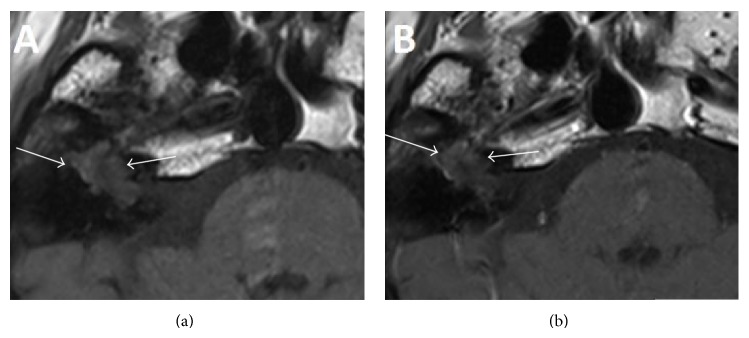
Pre- (a) and postcontrast (b) T1 weighted axial MRI imaging showing a largely isointense signal abnormality at the site of the mass seen on CT. There is no significant enhancement (*white arrows*).

A 63-year-old Caucasian male with a 10-year history of right-sided facial palsy and profound deafness was referred to our practice for further evaluation and management. Previous imaging had demonstrated a skull base lesion eroding the right petrous temporal bone. Computed tomography (CT) demonstrated that bone around the right geniculate ganglion and right cochlea was eroded with extension into the roof of the attic (Figure 1). The patient had previously consulted a neurologist who initially diagnosed Bell's palsy, later revising the diagnosis to a presumed vestibular schwannoma which was managed conservatively with serial magnetic resonance imaging (MRI). However, the mass on MRI was isointense to brain and showed no enhancement suggesting that a vestibular schwannoma was unlikely (Figure 2). The patient had been monitored by serial imaging for 4 years before electing for reconstructive surgical management to improve his appearance. Following two unsuccessful rhytidectomies and a static procedure for cosmesis, the patient was referred for review to our centre.

**Figure 3:**
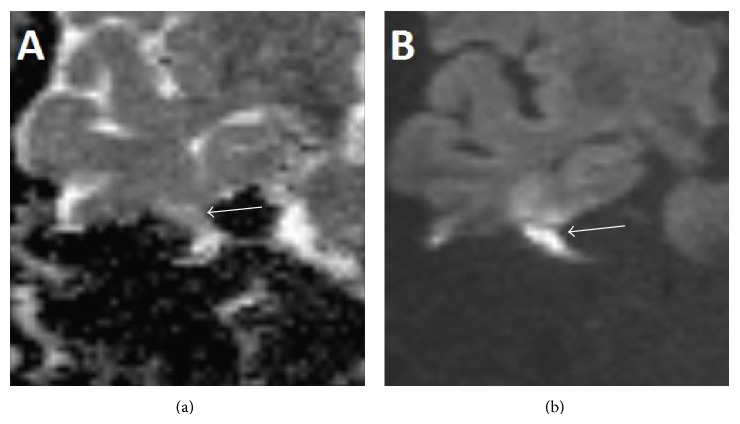
ADC imaging (a) and diffusion imaging (b) demonstrating restricted diffusion of lesion (*white arrows*).

Clinically the patient had a House-Brackmann grade 6/6 palsy [6], and there was evidence of extensive scarring from previous reconstructive surgery to the face, right ear, and neck. Otoscopy demonstrated an attic cholesteatoma. The drum was intact. Given that the patient had already developed damage to the inner ear and facial palsy, the initial decision was to manage him conservatively. However, this was later revised after the patient started having epileptic fits. Imaging review (Figures 1 and 2) alongside further diffusion weighted MRI showing a lesion with restricted diffusion meant that a congenital cholesteatoma was more likely (Figure 3). A facial nerve neuroma had been considered due to the location of the lesion but the lack of enhancement on MRI was against this diagnosis.

The initial surgical plan had been to approach the lesion via either a transcochlear or transotic approach. However, concerns relating to potential wound healing problems due to scarring from previous facial reconstructive procedures allied with the patient's poor health status necessitated a wholly endoscopic approach.

## 3. Description of Procedure

A 4 mm endoscope with a Storz video camera system was used along with a standard middle ear surgery set. The whole of the tympanic membrane was removed along with a 4 mm cuff of ear canal skin. The incudostapedial joint was disarticulated before removing the incus and malleus. The lateral wall of the attic was then curetted away to expose the cholesteatoma which had eroded the tympanic fallopian canal, lateral and superior canals, internal auditory meatus, geniculate ganglion, and epitympanic recess. The cholesteatoma was abutting, but not eroding the bone covering the horizontal portion of the carotid artery. It was removed in its entirety. A CSF leak was initially managed with controlled suction, pressure with neurosurgical pate, and subsequently with fat harvested from the thigh. It is important to stress that the CSF leak was an anticipated complication of surgery owing to the extent and location of the cholesteatoma and the extensive damage to the surrounding tissue as a result of previous surgical procedures.

The rest of the middle ear space and Eustachian tube opening was obliterated using fat, and fascia lata was used to fashion a drum graft. A lumbar drain was left in situ for three days and the patient was discharged on day five postoperatively. A subsequent diffusion weighted imaging sequence MRI did not show any residual disease. The patient will be monitored with serial DWI MRI imaging to detect any potential recurrence. He is disease-free at 9 months.

## 4. Discussion

The surgical objective in the management of a cholesteatoma of the petrous apex is complete surgical excision whilst avoiding both damage to neuronal tissue and a cerebrospinal fluid leak [1, 2]. However, this remains challenging because of difficulties in attaining adequate vision of the operative field which inevitably has an impact on recurrence [1, 2, 5]. Complete excision is further complicated by the ability of the thin matrix membrane of the cholesteatoma to adhere to other vital structures such as the dura, the internal carotid artery, and the jugular bulb [1, 7]. The lack of adequate exposure and the ability of the matrix membrane to adhere to other structures can lead to incomplete excision of the lesion thereby complicating management still further particularly where fat and muscle are used to eradicate the petrous cavity [1, 2, 7]. In cases of incomplete excision, the fat and muscle used to eradicate the petrous cavity will consolidate with residual lesion making it difficult to remove [7, 8].

The surgical approach to be adopted is dependent on the location of the lesion, the anatomical orientation of the internal carotid canal and the jugular bulb, and whether hearing has been affected [1, 2, 8]. The aim of the surgical approach is to provide clear views of the middle and posterior fossa, dura, carotid artery, lateral sinus and jugular bulb, and facial nerve notwithstanding the complexity of the anatomy [1, 2]. Unfortunately, traditional approaches to the petrous apex and surrounding areas mainly provide restricted access and view of the important structures, with the ear canal only being used to access the anterior apex even with a postauricular tympanomastoidectomy [5]. This in turn leads to poor views of other vital structures such as the sinus tympani and hypotympanum even when the facial nerve is mobilised and displaced [5]. Furthermore, a permeatal approach using a microscope is technically difficult and is inherently limited by the narrowest part of the ear canal [5].

A wholly endoscopic permeatal approach circumvents some of the problems encountered in microscopic surgery. It provides a better operative field and excellent vision of the important structures because, unlike the microscope, it bypasses the narrowest point and provides an excellent appreciation of the surrounding structures [5, 9]. A further observation in support of a wholly endoscopic permeatal approach is that it provides a more direct access to the apex with the scutum being the only structure between the apex and the endoscope. Removal of the scutum, as was performed in this case, further increases the field of vision [5]. As demonstrated in our case and elsewhere, the endoscope allows the surgeon to visualise and pass instruments like drills and curettes allowing better visualization of structures that are parallel to the axis of the microscope [5].

A wholly endoscopic permeatal procedure can also be used for removal of lesions in the internal ear canal fundus, intracochlear, intravestibular, and pericarotid regions [9]. It has to be said that the technique is hampered by being a one-handed technique and instrument design for endoscopic ear surgery is still evolving. However, the superior view obtained with endoscopes as well as prior experience of doing endoscopic surgery, particularly in the nose, helps circumvent these drawbacks. It is probably not worth using an exclusive permeatal approach where the disease process involves the mastoid [9]. Further, in cases where hearing has not been affected, other approaches might be more advantageous than the approach we describe in this report.

## 5. Conclusion

A wholly endoscopic permeatal approach to the petrous apex as we have described above can be used safely in the management of petrous apex cholesteatomas. In addition to offering excellent views and better access to the operative field, this minimally invasive technique causes less trauma to normal tissues and reduces postoperative morbidity and hospital stay. This case also highlights the importance of considering temporal bone pathology when investigating facial palsy.
